# Sharing and giving across adolescence: an experimental study examining the development of prosocial behavior

**DOI:** 10.3389/fpsyg.2014.00291

**Published:** 2014-04-11

**Authors:** Berna Güroğlu, Wouter van den Bos, Eveline A. Crone

**Affiliations:** ^1^Institute of Psychology, Leiden UniversityLeiden, Netherlands; ^2^Center for Adaptive Rationality, Max-Planck-Institute for Human DevelopmentBerlin, Germany

**Keywords:** friendship, prosocial behavior, fairness, trust, reciprocity, adolescence, peer relationships

## Abstract

In this study we use economic exchange games to examine the development of prosocial behavior in the form of sharing and giving in social interactions with peers across adolescence. Participants from four age groups (9-, 12-, 15-, and 18-year-olds, total *N* = 119) played three types of distribution games and the Trust game with four different interaction partners: friends, antagonists, neutral classmates, and anonymous peers. Nine- and 12-year-olds showed similar levels of prosocial behavior to all interaction partners, whereas older adolescents showed increasing differentiation in prosocial behavior depending on the relation with peers, with most prosocial behavior toward friends. The age related increase in non-costly prosocial behavior toward friends was mediated by self-reported perspective-taking skills. Current findings extend existing evidence on the developmental patterns of fairness considerations from childhood into late adolescence. Together, we show that adolescents are increasingly better at incorporating social context into decision-making. Our findings further highlight the role of friendships as a significant social context for the development of prosocial behavior in early adolescence.

## Introduction

Prosocial behavior, defined as voluntary behavior intended to benefit others (Eisenberg et al., 2006), plays a key role in social interactions. Displays of prosocial behavior strengthen future ties between individuals and are crucial for the formation and continuation of relationships (Fehr et al., 2002). Although most studies have examined interactions with anonymous others, the majority of our social interactions are with people we know. Social behavior depends heavily on the relation we have with our interaction partners, such that prosocial behavior (including displays of fairness, trust, and reciprocity) is employed based on past experiences with the interaction partner and the prospect of future interactions (Burnham et al., 2000; Delgado et al., 2005; van den Bos et al., 2011a). This raises the question how prosocial behavior in these anonymous games reflects, or differs from, social behavior toward familiar peers. From a developmental perspective, the role of peer relationships in social interactions is an intriguing question given that with age there is a growing focus on peers, and that by adolescence individuals spend the majority of their time with them (Brown, 2004). As such, the peer group has been identified as one of the most significant developmental contexts with profound effects on the development of prosocial behavior (Carlo et al., 1999). This paper aimed to specifically examine the development of sharing and giving as observed in fairness- and trust-related social decisions when interacting with peers.

Prosocial behavior in the form of sharing and giving typically involves making decisions involving consequences for others and is based on comparisons of outcomes for self and others. These behaviors have been examined using different sorts of allocation games, which typically involve the distribution of resources between two players (Rilling and Sanfey, 2011). In these games with varying rules, the first player (i.e., the proposer) is typically asked to make a decision (i.e., an offer) on how to divide the stake between him/herself and a second player (i.e., the responder). In the current study, we focused on two types of allocation games that are specifically well-suited to study prosocial behavior in the form of sharing and giving.

The first type involves a set of allocation games developed to study fairness considerations, which refer to the direct comparison of outcomes for self and other (Fehr et al., 2008). In these games, the players are asked to choose between a fair distribution of goods (e.g., coins) with equal pay-offs to both players and an alternative unfair distribution that might be advantageous or disadvantageous for the self. Using these games with differing alternative distributions it is possible to systematically examine the role of costs to the self in sharing and giving. Prosocial responding assessed by such experimental paradigms is already shown in two and a half-year-old children, whose behavior is not contingent on prosocial or selfish behavior of their interaction partners (Sebastián-Enesco et al., 2013). Already by 3 years of age, children have an understanding of the fairness norm and that others expect them to share equally (Smith et al., 2013). Fehr et al. (2008) demonstrated that there is an increase in the preference for fair (or equal) splits between age 3 and 8 years. This finding is in line with prior studies with varying allocation paradigms showing that equity preferences increase across early childhood, even at the cost of throwing away resources (Blake and McAuliffe, 2011; Shaw and Olson, 2012). Using a similar choice-card task where participants could choose between different allocations of points for themselves and friends, Berndt (1985) has also shown an age related increase in preferences for equal distributions over competition between 10 and 14 years of age. Recently, Steinbeis and Singer (2013) have provided further support for the developmental pattern of age related increase in these equity (fairness) preferences between the age of 7 and 13 years.

Despite the general trend of age related increase in fairness preferences (as assessed by relative number of fair/equal splits chosen) across these different games, differences in these preferences based on context have also been demonstrated. For example, both Fehr et al. (2008) and Steinbeis and Singer (2013) have shown that the preference for equal distributions was lower when they were costly than when they did not incur costs for the self. Further, age differences in choosing fair distributions were less pronounced when these choices were not costly than when they incurred costs. These findings suggest that the preference for fairness is dependent on the context regarding available alternatives. In the current study, we aimed to further examine these context effects in fairness related prosocial behavior in relation to different interaction partners.

A second sort of allocation paradigm suitable for examining sharing and giving is the Trust game (Berg et al., 1995). Trust behavior refers to decisions that favor other-regarding outcomes with the hope of future cooperation and self-gain (Larson, 1992). Reciprocity, such as returning a favor, refers to mutual exchange and is crucial for maintaining positive interactions (Lahno, 1995). In the Trust game, a first player can trust a second player to divide a stake, and the second player's reciprocity is an index for returning the favor initiated by the first player. In this sense, the trust choice assesses the extent of willingness to share and reciprocity assesses giving back. Interestingly, in these studies prosocial behavior, as indexed by level of trust and reciprocity, is even observed in one-shot social interactions with anonymous others where there is no prospect of future interactions between the two players. Developmental studies with the Trust game suggest that there are age related increases in trust and reciprocity toward anonymous others (Sutter and Kocher, 2007; van den Bos et al., 2010).

A social information processing approach has proven valuable to understanding the development of prosocial behavior. Prosocial young adolescents are shown to hold benign attributions, prefer to maintain a positive relationship with aggressive provocateurs, and show less negative emotionality in interactions (Nelson and Crick, 1999). Several studies have specifically focused on the role of dyadic characteristics in social behavior, showing that social-cognitive evaluations and behaviors are specific for interaction partners (Card and Hodges, 2007). Accordingly, interaction partners can evoke emotions that influence perception as well as processing of information, which together determine the behavioral output in context. For example, 4-year-olds attribute different emotions to the target depending on whether the target is a friend or a neutral classmate and are also more ready to help the target if the target is a friend. In adolescence, hostile attribution errors toward a specific peer are related to reactive aggression perceived from that peer (Hubbard et al., 2001; see also Ray and Cohen, 1997; Peets et al., 2007; Nummenmaa et al., 2008). In the current study we took a dyadic perspective in examining social behavior in the peer relationship context and we specifically expected that peer relations crucially influence displays of prosocial behavior.

In the current study we investigated how prosocial behavior is influenced by peer relationships by combining allocation games with sociometric mapping of relationships within across a wide age range of 9 to 18 years. Participants played a set of three allocation games (Fehr et al., 2008) and a Trust game (Berg et al., 1995) with four interaction partners: friends, antagonists, neutral peers, and anonymous peers. Based on prior studies using one-shot interactions (Sutter, 2007; Güroğlu et al., 2009b; van den Bos et al., 2010), we expected that in the current study participants would show increasing levels of prosocial behavior (defined as choices maximizing other's outcome) with increasing age.

A number of studies with varying methodology, paradigms, and measures have shown that children treat friends and non-friends differently. There is evidence for this differential treatment of in-group members (classmates/friends) vs. out-group members (anonymous peers/strangers) already by age three or four (Costin and Jones, 1992; Fehr et al., 2008; Moore, 2009), also when children are interacting with a doll protagonist (Olson and Spelke, 2008). Similarly, 3-year-olds are shown to share equally with collaborators (Warneken et al., 2011) and 5-year-old children display strong ingroup preferences with random group assignment and lack of a competitive context, both in terms of implicit and explicit attitudes, as well as resource allocation (Dunham et al., 2011). Some studies show a further differentiation between familiar peers. Examining reward allocations and helping behavior, Berndt (1985) has shown that young adolescents treat interaction partners differentially: adolescents were more generous and helping toward friends than toward neutral classmates. Similarly, Buhrmester et al. (1992) have shown that children and adolescents share more with friends than with neutral peers and share least with disliked peers; Amato (1990) has also shown that young adults help friends more than they help strangers. In the current study, we aimed to move beyond a dichotomous exploration of ingroup vs. outgroup members and examine peer relationships with varying valence (positive, negative, and neutral) and compared to unfamiliar peers. Furthermore, the majority of these previous studies have examined early childhood, whereas less is known about the changes in social decision-making across adolescence. In the current study, we focus on a broad age range across middle childhood and adolescence (9- to 18-year olds) where we can assess peer relationships in a structured environment, i.e., the classroom, using the same methodology, i.e., sociometric nominations. We expected that prosocial behavior would be moderated by the interaction partner, where participants were expected to display highest levels of prosocial behavior toward friends and lowest levels toward antagonists. We also expected this differentiation to be modulated by the specific allocation game.

It has further been shown that young adolescents become more relationship-focused with age, as indicated by more relational attributions to provocations from peers (Nelson and Crick, 1999). This is in line with the theoretical perspectives in changes in interpersonal interactions in general, and in friendships in particular, across adolescence (Selman, 1980). The development of cognitive skills and perspective-taking across adolescence are central to Selman's theory of interpersonal growth. Previous findings showing that older adolescents are increasingly better able to incorporate context related information into their decision-making process are further in line with these theoretical perspectives (Güroğlu et al., 2009a,b). Along similar lines, Berndt (1985) has shown that 14-year-olds differentiate more between friends and neutral classmates than 10- and 12-year-olds in displays of generosity. Such findings are also supported by studies examining the development of friendships. Around late childhood and early adolescence there is a specific increase in prosocial behavior such as helping and sharing as well as a concern for equality in interactions with friends (Youniss, 1980; Berndt, 1981; Furman and Bierman, 1984). This age related difference on the increasing specificity of friends was expected to reflect in age related differences in prosocial behavior toward friends in the current study. Taken together, we expected the moderation by interaction partner in prosocial behavior levels to be more pronounced for older participants than for younger ones.

One of the mechanisms that may account for developmental differences in prosocial behavior is the ability to take the other player's perspective. From a developmental perspective, the cognitive ability of role taking has implications for the development of altruistic motivation and behavior (Hoffman, 1975). Experimental studies in children as young as 3–4 years old show links between theory of mind skills and future-oriented prosocial behavior (Moore et al., 1998). A positive relation between prosocial behavior and perspective-taking skills has long been established (Eisenberg and Miller, 1987; Eisenberg et al., 1991; Carlo and Randall, 2002). It has been suggested that the components that are related to the consistency of prosocial behavior across time are related to, besides temperamental/genetic predispositions, inhibitory control and “other-orientation” (Eisenberg et al., 1999). This component of “other-orientation” is tapped by the cognitive ability to take others' perspectives and incorporate these perspectives into decision-making, which continues to develop into late adolescence (Dumontheil et al., 2009). The development of this ability of perspective-taking in social settings has been suggested to be a mediator of the development of prosocial behavior with increasing age (Iannotti, 1985). In prior studies we demonstrated the role of perspective taking by correlating the self-report index of the Interpersonal Reactivity Index (IRI, Davis, 1983) with prosocial behavior (Overgaauw et al., 2012), as well as a relation between affective perspective taking and prosocial behavior in the form of costly compensation of victims (Will et al., 2013). In the current study, we tested for the mediating role of perspective-taking skills in the development of prosocial behavior. We expected that the age related increase in prosocial behavior in both the set of allocation games and the Trust game would be more pronounced for individuals with higher levels of self-reported perspective taking.

## Methods

### Participants

A total of 125 participants took part in the study. The majority of the participants (90.4%) were Dutch, 2.4% was of Moroccan decent and 4.0% had another ethnic background; ethnic background information of four participants (3.2%) was missing. In order to control for the role of a general cognitive capacity, we assessed and controlled for IQ in our analyses. The pen-and-paper version of the Raven Standard Progressive Matrices (SPM) (Carpenter et al., 1990) was administered to assess an estimate of the participant's intelligence quotient (IQ). Due to time restrictions Raven scores of four participants were missing. After removing six outliers with IQ two standard deviations higher than the mean, estimate scores on IQ ranged between 94 and 130; the mean was 114.17 (*SD* = 9.37). The remaining 119 participants consisted of: 9-year-olds (*M* age = 9.27 years, *SD* = 0.53, 15 boys and 16 girls), 12-year-olds (*M* age = 11.89 years, *SD* = 0.64, 18 boys and 14 girls), 15-year-olds (*M* age = 15.07 years, *SD* = 050, 13 boys and 12 girls), and 18-year-olds (*M* age = 17.95 years, *SD* = 0.54, 8 boys and 23 girls). There were no differences in the gender distribution across age groups [χ^2^_(3)_ = 6.87, *p* = 0.08]. Thus, the sample sizes per age group ranged between 25 and 31, which is comparable to previous studies employing similar experimental designs (Fehr et al., 2008; Steinbeis and Singer, 2013).

There was a significant difference in IQ scores between the age groups [*F*_(3, 111)_ = 5.62, *p* = 0.001]. Tukey *post-hoc* tests showed that 18-year-olds had higher IQ (*M* = 119.65, *SD* = 8.37) than all other younger age groups (*M* = 112.50, *SD* = 10.37, *M* = 112.66, *SD* = 8.54, and *M* = 110.91, *SD* = 7.61, respectively for 9-, 12-, and 15-year-olds). Therefore, all analyses were run including IQ as a covariate; as suggested by Delaney and Maxwell (1981) the covariate was mean centered for ANCOVA analyses in a repeated measures design. There were no main effects of or interactions with IQ scores in any of the analyses reported below.

### Materials

#### Peer relationships

Friendship and antipathy relationships were identified based on sociometric nominations, and neutral peer relationships were based on peer ratings. Participants were provided with a numbered list of all classmates and were asked nominate up to five classmates for the questions “Who are your friends?” and “Who do you not like at all?” Mutual nominations on these items were used to identify friendship and antipathy relationships (i.e., positive and negative peer relationships), respectively (Güroğlu et al., 2007, 2009a). In addition, participants were asked to rate how much they liked each classmate on a scale ranging from (1) “do not like at all” to (3) “neither like nor dislike” to (5) “like very much.” Classmates who mutually gave a neutral rating (3) for one another were identified as neutral peer relationships.

#### Perspective-taking

Perspective-taking was measured by the Perspective-taking subscale of the IRI (Davis, 1983). This measure of perspective-taking was included because it (i) assesses the tendency to spontaneously adopt the psychological point of view of others (rather than e.g., a spatial point of view), (ii) assesses cognitive empathy skills (rather than e.g., affective empathy), (iii) is related to measures of interpersonal functioning, and (iv) is suitable for the broad age range of 9 to 18 years old. The perspective-taking subscale consisted of 6 items (e.g., “I try to look at everybody's side of a disagreement before I make a decision”) answered on a 5-point Likert scale ranging from (1) not true at all to (5) completely true. We used an adolescent version of the IRI, where items have been adapted for the youngest age group in the study. The scale had moderate reliability (Cronbach's alpha 0.68).

#### Fairness-related prosocial behavior

**Figure 1:**
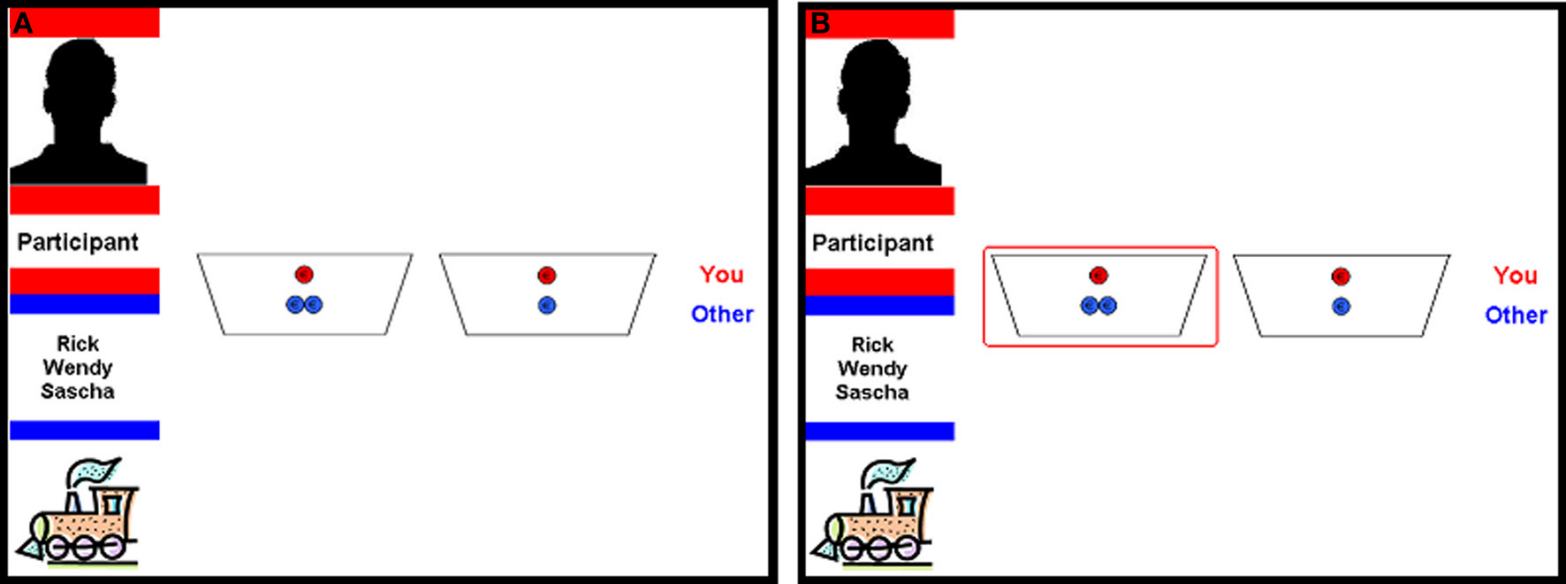
**Visual display for the allocation games. (A)** Two offers, each containing red and blue coins, indicate the share for the proposer and the interaction partner, respectively (here depicted *Disadvantageous prosocial* game 1/2 vs. 1/1). The left top panel displays the name of the proposer in red (here “Participant”). The left bottom panel displays the group (here group “train”) in the current trial and the names of the players in this group (here “Rick, Wendy, and Sascha”). **(B)** The red encircled option indicates the offer made by the participant.

A set of three allocation games were used to assess prosocial behavior related to fairness considerations (Fehr et al., 2008). Participants played these games on the computer where they were asked to distribute coins between themselves and their interaction partner by choosing one of the two preset distributions. One of the two options in each game was a fair distribution of coins with one coin for the self and one coin for the interaction partner [i.e., (1/1) distribution]. The alternative option varied between the three games, yielding three games: (i) the *Costly prosocial* game where the alternative option was two coins for self and zero coins for the other [i.e., (2/0) distribution], (ii) the *Non-costly prosocial* game where the alternative option was one coin for self and zero coins for the other [i.e., (1/0) distribution], (iii) the *Disadvantageous prosocial* game where the alternative option was one coin for the self and two coins for the other [i.e., (1/2) distribution] (see Figure 1). The dependent variable was the frequency of *prosocial* (i.e., not self-focused) choices [i.e., (1/1) distribution in the *Non-costly* and *Costly prosocial* games and (1/2) distribution in the *Disadvantageous prosocial* game] and was calculated separately per game and interaction partner.

Participants played a total of 48 trials of games in randomized order. The location of the fair distribution (1/1) was counterbalanced across trials. All three games were played four times with each of the four interaction partners (friends, antipathies, neutral, and anonymous peers). In order to render the games less repetitive and keep the participants engaged in these multiple trials, we used the following design: Participants were told that each round of the game would be played with one of the four groups that were predetermined by the researchers. They were explained that the peers in three of the four groups would be randomly chosen classmates and the fourth group would be anonymous same gender and age peers from another school. In fact, peers from the three groups with classmates were not randomly chosen classmates. Each of the three groups contained either friends, neutral peers, or antagonists identified based on the sociometric nominations and ratings obtained during the first data collection. In each group, there were one, two or three players.

Care was taken to present all four groups in a neutral manner so that participants would not be biased toward one group or another. To accomplish this, each group was randomly given one of the following neutral names: group Bike, group Car, group Airplane, and group Train. Participants were given lists of players in each group and were given ca. 5 min to study the group members. During each trial of the game, the list of players within a group was presented on the left side of the screen (see Figure 1). Each group was randomly assigned to the group of friends, antagonists, neutral classmates, and anonymous interaction partners.

Participants were told that they would play each trial with a single individual interaction partner from the group they were playing with but they would not know *exactly* with whom. This was done so that there would be no strategies for multiple distributions. It was further explained that the computer would keep track of their interaction partners in each trial in order calculate everyone's earnings, which would be paid out at the end of all trials. Each trial started with a fixation cross (1 s), followed by a screen presenting the group they are playing with (left panel) and the set of alternatives they could choose from. Participants had 5 s to respond by pressing a keyboard key. If they failed to respond within 5 s, a screen with “Too late!” was presented for 1 s. Upon response, their choice was encircled in red for 2 s and subsequently they were presented with the following trial. Completion of this task took about 2 min on average. Participants played six practice trials with the computer before the actual games started.

#### Trust-related prosocial behavior

**Figure 2:**
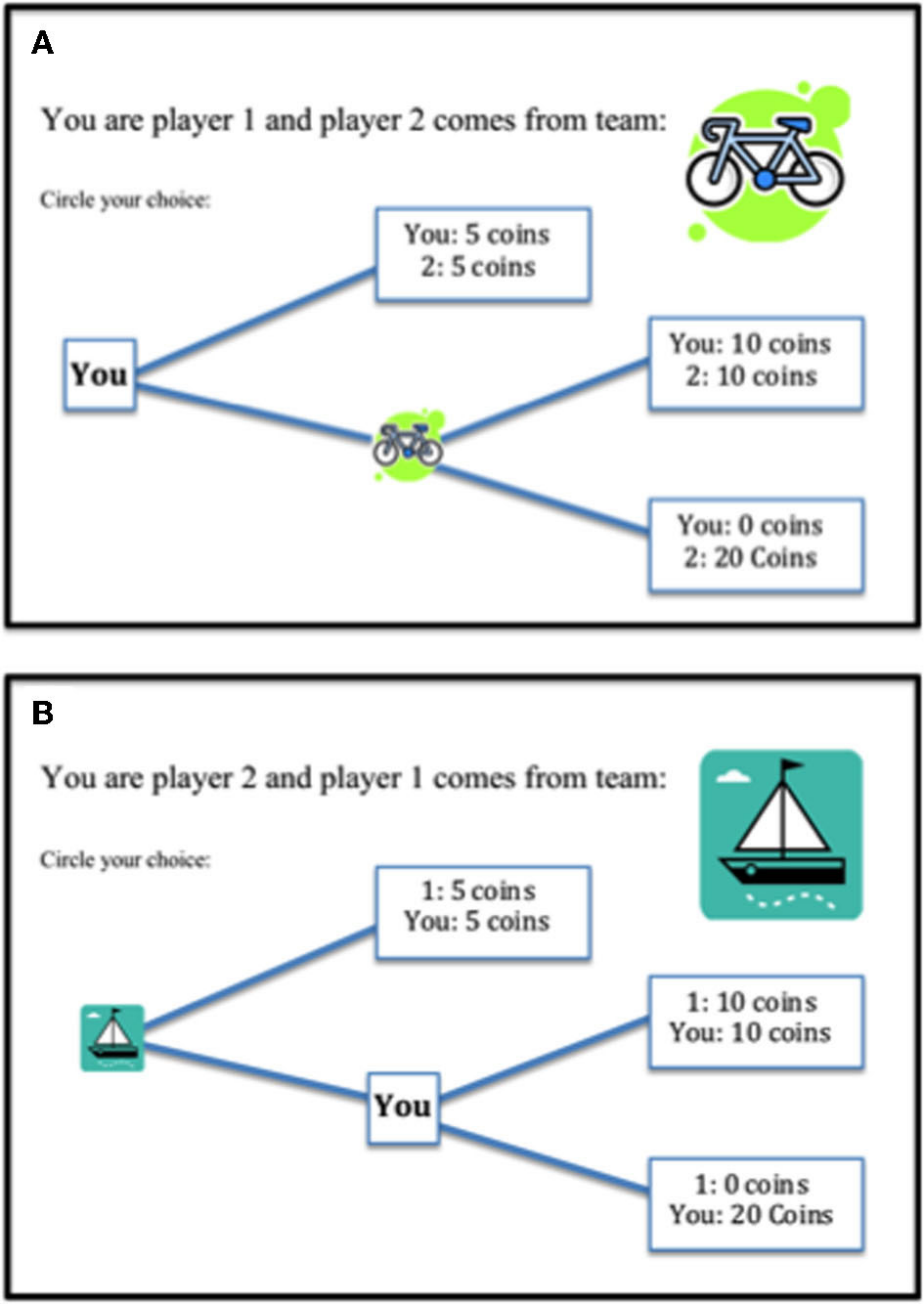
**Visual display for the Trust game. (A)** Participant is the investor (here “you”), the interaction partner is the trustee (here group “boot”). **(B)** Participant is the trustee (here “you”), the interaction partner is the investor (here group “bicycle”).

A single round of an adaptation of the Trust game (Berg et al., 1995) was used to assess trust and reciprocity in social interactions. Participants played the Trust game on paper once as the first player (investor) and once as the second player (trustee) with each of the four types of interaction partners (i.e., 8 rounds in total). The four interaction partners were presented in four groups in the same way as for the allocation games explained above. The starting stake was 10 coins and the first player could choose between two options: an equal distribution of 5 coins for self and 5 coins for the trustee, or letting the trustee decide (i.e., trust). In the latter case, the stake was doubled and the trustee had two options: give 10 coins each (i.e., reciprocate) or give nothing to the investor and take 20 coins for him/herself (i.e., defect). The options for the second player were visible to the first player from the start (see Figures 2A and 2B respectively for participant as investor and trustee). The dependent variable was the prosocial (i.e., not self-focused) choices made by the players and was coded in the following way: as the investor, the trust option was coded as 1 and no-trust option as 0; as the trustee, the reciprocate option was coded as 1 and defect option as 0. Average frequency of trust and reciprocity were calculated per age group and interaction partner.

### Procedure

Two elementary schools and one high school agreed to take part in the study. After consent was obtained from school authorities, informed consent was obtained from parents and teachers. The first part of the data collection was carried out in classrooms where participants filled out several questionnaires, including sociometric nominations and ratings for all classmates, perspective-taking scale and the pen-and-paper version of the Raven's SPM. This session lasted about 45 min. Approximately 1 week later at a second data collection day, computer tasks were presented on individual laptops with 15-inch monitors in a separate room. In groups of four at a time, participants completed the allocation tasks on the computer and the Trust game on paper. Care was taken that all instructions were clear. Previous studies have successfully employed similar experimental designs with computer based allocation and gambling games in the age groups assessed here (van Leijenhorst et al., 2008; Güroğlu et al., 2009a,b; van den Bos et al., 2010). This session lasted for about 30 min. At both data collection points participants were explained that their participation was voluntary and were ensured that their responses would remain anonymous. In order to further assure anonymity, we also emphasized during the second data collection point that the computer tasks were not online interactions and that classmates could not see the participants' responses. We also took care to place individual laptop computers facing away from each other so that it was not possible for the participants to view each other others' responses.

Participants were told that the coins in the allocation tasks were valuable. It was explained that after all participants completed the allocation tasks and data collection was completed each participant would be paid a randomly chosen number of trials. It was emphasized that their decisions would determine the earnings for themselves as well as for their interaction partners. After data collection was completed, in agreement with the schools and parents all participants were paid a fixed amount of 3 euros (~5 US dollars) each. This procedure was approved by the local ethics committee.

## Results

### Manipulation check

At the end of the second session participants were asked to make a list of players in each group and were asked to indicate what they thought of each group (except for the group with anonymous players). This was assessed as a manipulation check to ensure that the participants paid attention to the group members and that they distinguished between the three groups of classmates each containing friends, antagonists, and neutral classmates in terms of likeability. Percentage of correct recall for the players in each group was high (*M* = 82%, *SD* = 20%). Fifteen-year-olds recalled significantly more players than 12-year-olds [*M* = 91 and 75%, respectively; *F*_(3, 104)_ = 3.53, *p* = 0.02]. Participants recalled players from the friend group (91%) more often than players from the antagonist (82%) and neutral peer groups (79%) [*F*_(1, 91)_ = 13.0, *p* < 0.001 and *F*_(1, 95)_ = 16.8, *p* < 0.001, respectively]. Open-ended questions on what the participants thought of each group were recorded on a five-point scale ranging from (1) very negative to (5) very positive. Participants rated the friend group (*M* = 4.98, *SD* = 0.06) more positive than the neutral group [*M* = 3.84, *SD* = 0.16; *F*_(1, 99)_ = 47.7, *p* < 0.001], which was rated more positively than the antagonist group [*M* = 3.26, *SD* = 0.23; *F*_(1, 94)_ = 4.90, *p* = 0.03]. This manipulation check confirmed our expectation that the participants differentiated between different groups in terms of their relationships with them. The ratings for each group did not differ across the age groups [*F*_(3, 88)_ = 0.92, *p* = 0.44, η^2^_*P*_ = 0.03]; there was also no age group × group interaction in the ratings [*F*_(5.27, 154.64)_ = 1.19, *p* = 0.32, η^2^_*P*_ = 0.04].

### Descriptives

#### Peer relationships

The mean number of mutual friendships, antipathies, and neutral relationships were 2.75 (*SD* = 1.62), 0.36 (*SD* = 0.76), and 5.26 (*SD* = 4.51), respectively. Univariate analyses of variance (ANOVA) with age group as the between subjects factors yielded a main effect of age for number of friendships and antipathies [*F*_(3, 113)_ = 2.75, *p* = 0.05, η^2^_*P*_ = 0.07 and *F*_(3, 113)_ = 3.54, *p* = 0.02, η^2^_*P*_ = 0.09, respectively]. There were more friendships in 15-year-olds (*M* = 3.43, *SD* = 1.50) than in 18-year-olds (*M* = 2.26, *SD* = 1.32). Nine-year-olds (*M* = 0.71, *SD* = 1.07) had more antipathy relationships than 18-year-olds (*M* = 0.13, *SD* = 0.34).

#### Perspective-taking

The perspective-taking scores ranged from 1.17 to 4.83 with a mean of 3.32 (*SD* = 0.63). There was a significant correlation between perspective-taking and age [*r*_(117)_ = 0.35, *p* < 0.001] and between perspective-taking and IQ [*r*_(113)_ = 0.23, *p* = 0.02]; the correlation between age and perspective-taking remained significant when controlling for IQ [partial *r*_(110)_ = 0.34, *p* < 0.001].

### Prosocial behavior in fairness considerations

A repeated measures analysis of variance with Age group (four levels: 9-, 12-, 15-, and 18-year-ols) as the between subject factors and Relationship type (four levels: friends, antipathies, neutral peers, and anonymous peers) as the within subject factor was conducted for frequency of prosocial offers made in each of the three games^1^. All analyses where the Mauchly's test indicated a violation of the assumption of sphericity, the Huyn-Feldt correction is reported.

**Figure 3:**
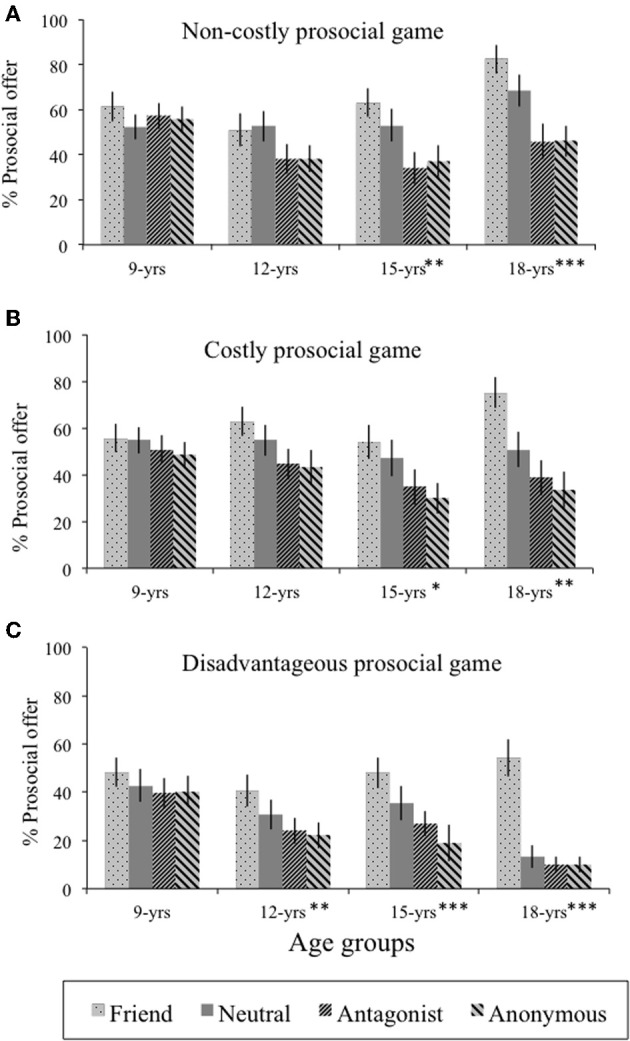
**Prosocial behavior in the allocation games**. Mean frequency (%) and standard errors of prosocial offers [i.e., (1/1) distribution in the **(A)** *Non-costly prosocial game* and the **(B)** *Costly prosocial game* and (1/2) distribution in the **(C)** *Disadvantageous prosocial game*] are presented per interaction partner for the four age groups. Age differences are indicated by an asterisk (^*^). ^*^p < 0.05, ^**^p < 0.01, ^***^p < 0.001.

In the *Non-costly prosocial* game (see Figure 3A), participants chose the prosocial offer [i.e., (1/1) distribution] on 52% of the trials (*SD* = 29%). The main effect of Age group was not significant [*F*_(3, 103)_ = 2.34, *p* = 0.08, η^2^_*p*_ = 0.06]. There was a main effect of Relationship type [*F*_(3, 309)_ = 16.5, *p* < 0.001, η^2^_*p*_ = 0.14]: prosocial behavior was higher for friends than for neutral peers [*F*_(1, 106)_ = 4.58, *p* = 0.04, η^2^_*p*_ = 0.04], which was again higher than for antagonists [*F*_(1, 106)_ = 13.6, *p* < 0.001, η^2^_*p*_ = 0.11]. Prosocial behavior toward antagonists and anonymous peers did not differ [*F*_(1, 106)_ = 0.04, *p* = 0.85]. This main effect was qualified by an Age group × Relationship type interaction [*F*_(9, 309)_ = 2.63, *p* = 0.006, η^2^_*p*_ = 0.07]. Nine- and 12-year-olds did not differ in their frequency of (1/1) offers across the four interaction partners [overall *M* = 57 and 45%, *SD* = 26% and 28%, *F*_(2.41, 48.3)_ = 0.69, *p* = 0.56, η^2^_*p*_ = 0.02 and *F*_(3, 81)_ = 1.76, *p* = 0.16, η^2^_*p*_ = 0.06, respectively]. In contrast, 15- and 18-year-olds differentiated in their responses toward the other players [*F*_(2.41, 48.3)_ = 5.26, *p* = 0.006, η^2^_*p*_ = 0.21 and *F*_(3, 75)_ = 6.22, *p* = 0.001, η^2^_*p*_ = 0.20, respectively]. Tukey *post-hoc* tests indicated that 15- and 18-year-olds were more prosocial toward friends (*M* = 63 and 82%, respectively) than toward antagonists and anonymous peers (*M* = 37 and 46% anonymous peers, and *M* = 34 and 46% antagonists, respectively for 15- and 18-year-olds; all *F* > 6.21, *p* < 0.02). Further, both 15- and 18-year-olds displayed more prosocial behavior toward the neutral peers (*M* = 53 and 69%, respectively) than toward antagonists (*M* = 34 and 46%, respectively; all *F* > 4.90, *p* < 0.04).

In the *Costly prosocial* game (see Figure 3B), participants chose the fair (1/1) distribution on approximately 50% of the trials (*SD* = 29%). The main effect of Age group was not significant [*F*_(3, 103)_ = 0.9, *p* = 0.47, η^2^_*p*_ = 0.03]. There was a main effect of Relationship type [*F*_(2.87, 296)_ = 18.7, *p* < 0.001, η^2^_*p*_ = 0.15]. As in the *Non-costly prosocial* game, prosocial behavior was again higher for friends than for neutral peers [*F*_(1, 106)_ = 9.07, *p* = 0.003, η^2^_*p*_ = 0.08], which was again higher than for antagonists [*F*_(1, 106)_ = 13, *p* < 0.001, η^2^_*p*_ = 0.11]; prosocial behavior toward antagonists and anonymous peers again did not differ [*F*_(1, 106)_ = 0.28, *p* = 0.60]. This interaction was qualified by an Age group × Relationship type interaction [*F*_(8.62, 296)_ = 2.33, *p* = 0.02, η^2^_*p*_ = 0.06]. Again, 9- and 12-year-olds did not differ in their frequency of prosocial offers across their interaction partners [overall *M* = 55 and 51%, *SD* = 29 and 27%; *F*_(2.58, 72.2)_ = 0.92, *p* = 0.42 and *F*_(3, 81)_ = 2.53, *p* = 0.06, respectively]. For the other two age groups, a differentiation was observed [*F*_(3, 60)_ = 3.53, *p* = 0.02, η^2^_*p*_ = 0.15 and *F*_(3, 75)_ = 4.84, *p* = 0.004, η^2^_*p*_ = 0.16, for 15- and 18-year-olds, respectively]: participants displayed more prosocial behavior toward friends (15-year olds *M* = 57%; 18-year-olds *M* = 75%) than toward anonymous peers (15-year-olds *M* = 28%; 18-year-olds *M* = 34%; all *F* > 4.99, *p* < 0.04) and antagonists (15-year-olds *M* = 34%; 18-year-olds *M* = 39%; all *F* > 7.58, *p* < 0.01). Furthermore, 18-year-olds were also more prosocial toward their friends than toward neutral peers [*M* = 51%, *F*_(1, 25)_ = 10.2, *p* = 0.004, η^2^_*p*_ = 0.29].

Finally, in the *Disadvantageous prosocial* game (see Figure 3C), the prosocial (1/2) distribution was chosen on approximately one-third of the trials (*M* = 32%, *SD* = 26%). The main effect of Age group was not significant [*F*_(3, 98)_ = 2.56, *p* = 0.06, η^2^_*p*_ = 0.07]. There was again a main effect of Relationship type [*F*_(2.57, 252)_ = 26.2, *p* < 0.001, η^2^_*p*_ = 0.21]. Prosocial behavior was higher for friends than for neutral peers [*F*_(1, 101)_ > 20.7, *p* < 0.001, η^2^_*p*_ = 0.17]. Behavior toward neutral peers, antagonists, and anonymous peers did not differ significantly [*F*_(1, 101)_ = 3.70, *p* = 0.06, η^2^_*p*_ = 0.04]. There was also a significant Age group × Relationship type interaction in the *Disadvantageous prosocial* game [*F*_(7.71, 252)_ = 2.36, *p* = 0.02, η^2^_*P*_ = 0.07]. As in the *Non-costly prosocial* game and the *Costly prosocial* game, 9-year-olds did not differ in frequency of prosocial choices across interaction partners [overall *M* = 43%, *SD* = 26%, *F*_(1, 28)_ = 1.50, *p* = 0.23]. In contrast, 12-, 15-, and 18-year-olds were more prosocial toward their friends (*M* = 41%, *M* = 48%, and *M* = 54%, respectively; all *F* > 7.81, *p* < 0.01) than toward antagonists (*M* = 24%, *M* = 27%, and *M* = 10%, respectively) and anonymous peers (*M* = 22%, *M* = 19%, and *M* = 10%, respectively; all *F* > 9.90, *p* < 0.004). Both 15- and 18-year-olds displayed also more prosocial behavior toward their friends than toward neutral peers (*M* = 35% and *M* = 13%, respectively, all *F* > 6.38, *p* < 0.02).

### Prosocial behavior in trust and reciprocity considerations

**Figure 4:**
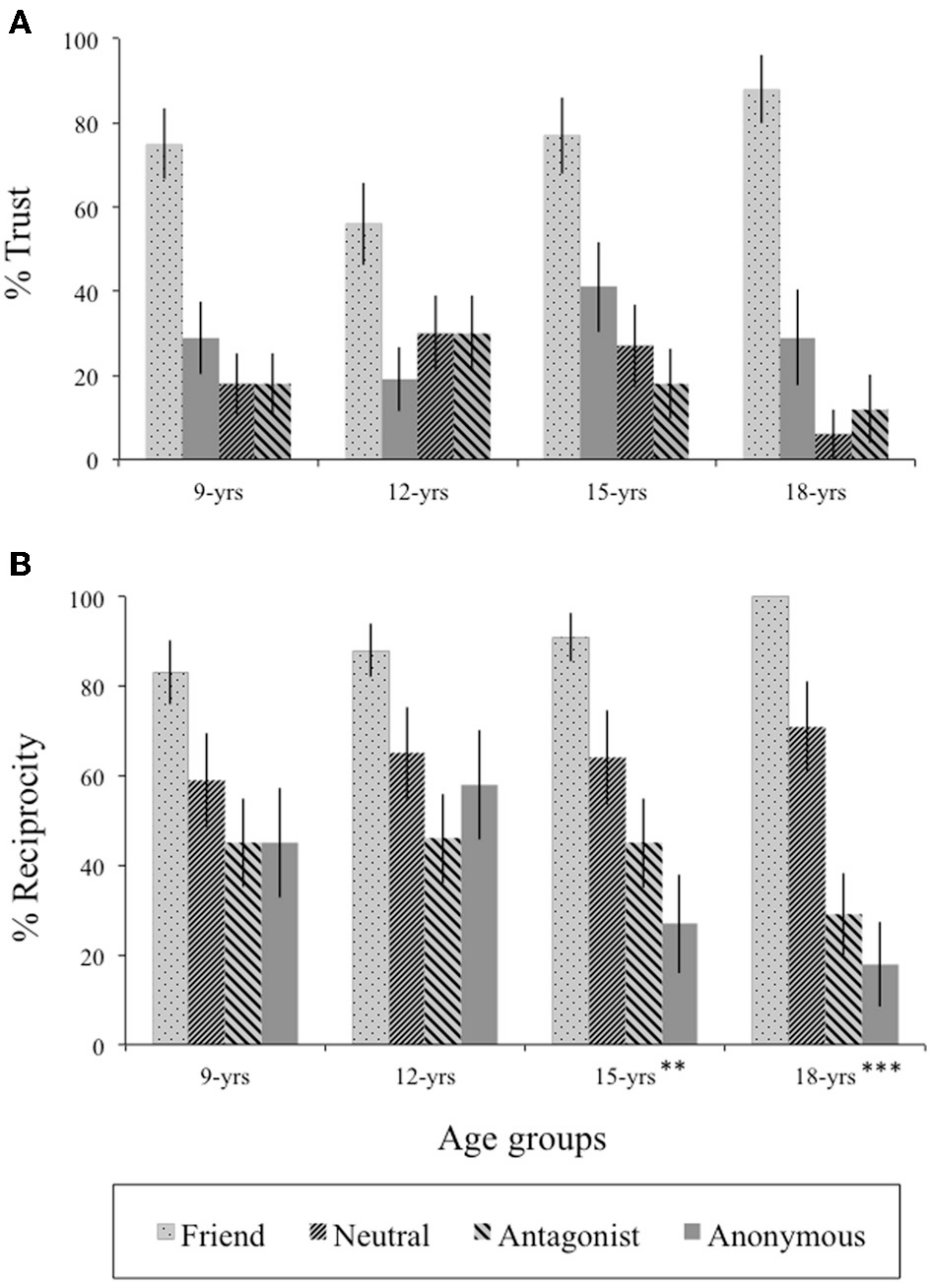
**Prosocial behavior (trust and reciprocity) in the Trust game**. Mean frequency (%) and standard errors of **(A)** trust and **(B)** reciprocity choices in the Trust game are presented per interaction partner for the four age groups. Age differences are indicated by an asterisk (^*^). ^**^p < 0.01, ^***^p < 0.001.

Two repeated measures analyses were conducted; one for trust and one for reciprocity choices with Age group as the between subjects factor and Relationship type as the within subject factor. For trust behavior (see Figure 4A), there was only a significant main effect of relationship [*F*_(3, 255)_ = 37.7, *p* < 0.001, η^2^_*P*_ = 0.31]. Participants trusted friends (*M* = 72%, *SD* = 45%) more often than other peers (all *F* > 61.7, *p* < 0.001). Trust displayed for antagonists (*M* = 21%, *SD* = 41%), anonymous (*M* = 20%, *SD* = 40%) and neutral peers (*M* = 29%, *SD* = 46%) did not differ from each other [*F*_(2, 170)_ = 2.40, *p* = 0.09]. There was no main effect of Age group or an interaction with Age group.

For reciprocity (see Figure 4B), there was a only main effect of Relationship, with higher reciprocity for friends than for other interaction partners [*F*_(3, 255)_ = 31.7, *p* < 0.001, η^2^ = 0.27]. Mean reciprocity ranged between 83% (9-year-olds, *SD* = 38%) and 100% (18-year-olds, *SD* = 0%). We examined the reciprocity scores for the other three interaction partners separately for the four age groups. These analyses showed that 9-, and 12-year-olds did not differ in reciprocity toward antagonists, neutral, and anonymous peers (all *F* < 2.80, *p* > 0.08). In contrast, 15- and 18-year-olds showed higher reciprocity toward neutral peers (*M* = 63%, *SD* = 50% and *M* = 68%, *SD* = 48%, respectively) than toward anonymous peers [*M* = 26%, *SD* = 45% and *M* = 20%, *SD* = 41%; *F*_(1, 17)_ = 8.01, *p* = 0.01, η^2^ = 0.32 and *F*_(1, 23)_ = 9.50, *p* = 0.005, η^2^_*P*_ = 0.29, respectively for 15- and 18-year olds].

### Mediating role of perspective-taking

Next, we investigated the mediating role of perspective-taking in the link between age and prosocial behavior. For this purpose, we followed the mediator analysis and SPSS syntax provided by Preacher and Hayes (2004). This method tests whether an indirect effect (i.e., the path from age to prosocial behavior with perspective-taking as mediator) is significantly different from zero. Accordingly, we examined the coefficients for (*a*) the link between the independent variable (i.e., age) and the mediator (i.e., perspective-taking), and (*b*) the link between the mediator (i.e., perspective-taking). We used a bootstrapping technique with 10,000 iterations and computed the 95% confidence interval around the product term a^*^b. The mediation effect is significant if zero falls out of this confidence interval. Considering that the direct effect of age on prosocial behavior is a prerequisite for testing mediation, we focused our analyses on those dependent variables where we observed a significant correlation with age: prosocial behavior with friends and neutral peers in the *Non-costly prosocial* game [*r*_(105)_ = 0.21, *p* = 0.03 and *r*_(105)_ = 0.19, *p* = 0.05, respectively], prosocial behavior with anonymous peers in the *Costly prosocial* game [*r*_(105) = −0.21_, *p* = 0.03], prosocial behavior with antagonists, neutral peers, and anonymous peers in the *Disadvantageous prosocial* game [*r*_(100) = −0.29_, *p* = 0.003, *r*_(100)_ = −0.27, *p* = 0.006, and *r*_(100)_ = −0.31, *p* = 0.002, respectively], and reciprocity with anonymous peers [*r*_(86) = −0.23_, *p* = 0.03].

**Figure 5:**
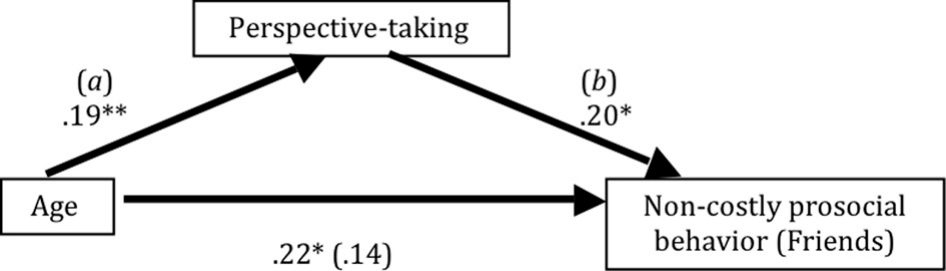
**Perspective-taking skills mediating the link between age and prosocial behavior**. Figure depicts the results of the mediation analyses and shows that perspective-taking is a mediator between age and non-costly prosocial behavior toward friends. ^**^p < 0.001, ^*^p < 0.05.

A significant mediation effect was found only for the *Non-costly prosocial* game with friends and not for the other dependent variables. The 95% confidence interval for the indirect effect ranged from 0.17 to 1.61, showing that perspective-taking mediates the direct link between age and prosocial behavior toward friends (see Figure 5). The direct effect of age on prosocial behavior was no longer significant when controlling for perspective-taking (β = 0.14), *t*_(111)_ = 1.72, *p* = 0.09.

## Discussion

The current study employed an experimental approach toward examining the development of prosocial behavior in social interactions with peers across adolescence. Our findings contribute to the existing literature examining context dependency of social behavior in three significant manners. First, we employed a variety of controlled experimental conditions examining forms of prosocial behavior such as costly and non-costly prosocial behavior, as well as trust and reciprocity, which provided us with different ways of assessing altruistic motivations aimed at maximizing outcomes for another person. Second, we examined behavior with four different interaction partners. Finally, we examined these processes across a wide age range from 9 to 18 years. More specifically, we demonstrated that 9- and 12-year-olds treated interaction partners similarly, whereas older adolescents' (15- and 18-years) prosocial behavior was significantly moderated by who their interaction partner was. Moreover, we demonstrated that perspective-taking skills mediated age related differences in prosocial behavior when interacting with friends.

### Development of prosocial behavior

We assessed prosocial behavior using a set of three allocation games: the *Non-costly prosocial* game, the *Costly prosocial* game, and the *Disadvantageous prosocial* game (Fehr et al., 2008). By presenting participants a dichotomous choice where one of the two options is always a (1/1) fair distribution, we were able to compare the preference for equal outcomes across different contexts. Three relevant processes need to be kept in mind in interpreting decision-making processes across these conditions: (1) a strong preference for equity, which would be indicated by equity choices (1/1) across games, (2) cost of choosing one distribution over the other in each game, and (3) payoff comparison for self vs. other, that is, whether the other gets more than self or not (Radke et al., 2012). A strong sense of equity requires participants to choose the (1/1) distribution regardless of context (i.e., game) with varying costs to the self.

Fehr et al. (2008) previously showed that prosocial behavior increases with age from 3 to 8 years, but that 8-year-olds have a stronger preference for equity, also when the alternative is a non-costly *and* prosocial distribution (i.e., in the *Disadvantageous prosocial* game). This result was replicated in the current study in adolescents. That is to say, overall levels of prosocial choices were lower in the *Disadvantageous prosocial* game than in the other two games, supporting context dependency of fairness considerations.

It is important to consider the current results in relation to previous findings. Although earlier findings have not been completely unanimous, several studies have shown no age differences in across 9 to 18 years in costly prosocial behavior assessed as fair allocations in a Dictator game (Gummerum et al., 2008; Güroğlu et al., 2009a,b). In the current study we also show that there are no age differences in the *Costly prosocial* game in interactions with classmates, whereas there is a slight age-related decline in fair allocations to anonymous others. Overall levels of prosocial behavior in the *Non-costly prosocial* game were somewhat lower than those reported by Fehr et al. (2008) for 7–8 year-olds (around 80%). However, Steinbeis and Singer (2013) reported equity choices in the *Non-costly prosocial* game to be around 15% for 7–8 year-olds, and around 60% for 11–13 year-olds, which is similar to our findings. In contrast, prosocial choices in the *Disadvantageous prosocial* game were higher in the current study than those reported previously, particularly for the youngest age group. As suggested by Steinbeis and Singer (2013), different incentives used in these studies form a plausible explanation for these discrepancies. Furthermore, previous studies examined interactions with anonymous others in general, whereas the current study introduced different interaction partners. It is likely that such differences in the experimental design shape choices, where participants' decisions are influenced by the broad context in which different decisions are being made across interaction partners (for a similar discussion, see (Güroğlu et al., 2009a,b). Interestingly, percentages of prosocial choices in the *Costly prosocial* game were comparable across all three studies. Future studies could investigate whether non-costly prosocial behavior is more sensitive to context factors than costly prosocial behavior.

In addition, we showed that interaction partners significantly moderated the developmental patterns of prosocial behavior across ages 9 to 18. Specifically, there was an age related increase in non-costly prosocial behavior (i.e., in the *Non-costly prosocial* game), but only toward friends and neutral peers. Costly prosocial behavior decreased with age toward anonymous peers. Thus, participants are willing to incur costs for an equitable distribution, but with increasing age less so for unknown others. Finally, in case of non-costly prosocial behavior that specifically benefits the other (i.e., in the *Disadvantageous prosocial* game), there was a decrease in the non-costly prosocial choices toward antagonists, neutral and anonymous peers. Thus, only for friends participants are willing to accept an unequal prosocial outcome. Such evidence for increasing as well as decreasing levels of prosocial behavior might help us better understand the previously reported contradictory findings on developmental patterns of prosocial behavior. Besides studies showing increasing levels of prosocial behavior (e.g., Eisenberg et al., 1991, 1995), there are findings suggesting a decline in prosocial behaviors from middle to late adolescence (e.g., Luengo Kanacri et al., 2013). Our findings suggest that future studies should better examine the role of interaction partners in displays of prosocial behavior to get a more nuanced idea on these developmental patterns.

The second set of analyses focused on trust and reciprocity in the Trust game. Contrary to expectations, trust- and reciprocity-related prosocial behavior showed no age related changes. That is to say, per interaction partner, participants of all ages showed similar levels of trust. Several prior developmental studies have demonstrated low levels of trust and reciprocity toward strangers in children and young adolescents, and that both trust and reciprocity behavior increase with age (Sutter and Kocher, 2007; van den Bos et al., 2010). The current findings add to this literature by showing that 9-year-olds can already display trust and reciprocity behavior when they are interacting with friends. Prior reports already indicated that interpersonal trust is an important aspect of friendship across childhood and adolescence (Bigelow and La Gaipa, 1975; Selman, 1980; Youniss, 1980). The reciprocal aspect of friendships increases in importance around elementary school and reciprocity remains to be the *deep structure* of friendships across the life-span (Hartup and Stevens, 1997).

### A closer look at the role of interaction partners in prosocial behavior

Young adolescents in the age group of 9- and 12-year-olds generally showed similar levels of prosocial behavior for all interaction partners. In contrast, 15- and 18-year-olds clearly differentiated in prosocial behavior depending on the interaction partner. When prosocial behavior was non-costly, 15- and 18-year-olds acted more prosocial toward friends and neutral peers than to disliked and anonymous ones; when it was costly, 18-year-olds further differentiated friends from neutral peers. Thus, the development of the differentiation of interaction partners in displays of costly prosocial behavior seems to be prolonged across adolescence; this might possibly be because prosocial behavior requires better control of self-outcome maximization.

Differentiation of interaction partners in displays of costly and non-costly prosocial behavior has been shown for 3.5- and 4.5-year-olds (Olson and Spelke, 2008; Moore, 2009). In light of these previous findings, it might be puzzling that 9- and 12-year-olds in our study did not differentiate at all between interaction partners. Our findings are further, however, in line with the findings of Buhrmester et al. (1992) where they show that 6- and 10-year-olds do not differentiate between friends and neutral peers in their sharing behavior, whereas 14-year-olds share more with friends than with neutral peers. The pattern of prosocial behavior of 15- and 18-year-olds in the current study, where we see the differentiation of friends from all other peers, fits well with the developmental role of friendships and their increasing importance across adolescence (Sullivan, 1953; Youniss, 1980).

The significant role of friendships across childhood and adolescence is further supported by the strong differentiation of friends from other peers in displays of trust and reciprocity. Participants of all ages showed highest levels of trust and reciprocity for friends. Oldest adolescents further differentiated between the other three peer groups, such that trust of anonymous and disliked peers were less often reciprocated than trust of neutral peers. It is noteworthy that even the youngest age groups differentiated between friends and other peers in their trust and reciprocity behavior, whereas this effect was lacking in the allocation games. It could be that trust and reciprocity develop initially within close relationships such as friendships, whereas fairness related prosocial behavior are more general forms of prosocial behavior that are not relationship-specific.

Interestingly, neither prosocial choices in the allocation games nor trust and reciprocity choices in the Trust game differed for disliked and anonymous peers in any of the age groups. It might be that within the current context both these groups were seen as an out-group and that adolescents differentiate mainly between in-group and out-group members of the peer group (Fehr et al., 2008). As Fehr et al. (2008) rightly indicate, prosocial behavior (particularly in the form of reciprocity) can be motivated by selfish impulses related to expectations of future benefits from interaction partners. In this respect, it could be that participants' lack of expectations to interact with disliked as well as with anonymous peers in the future might explain behavior in this context.

Taken together, across adolescence control of outcome-maximization and payoff comparisons are increasingly better incorporated into decision-making. These results are in line with our previous findings showing developmental patterns that are dependent on intentionality of unfair treatment (Güroğlu et al., 2009a,b, 2011; Overgaauw et al., 2012) and reputation based on previous interactions (Will et al., 2013). These findings show that social context information is increasingly better incorporated into decision-making. Prior studies showed that, despite stable individual differences, prosocial behavior is difficult to predict over time (Eisenberg et al., 1999). Our findings suggest that prosocial behavior is increasingly sensitive to factors related to the social context in which interactions take place, which might explain weak consistency in prosocial behavior in prior studies.

### Role of perspective-taking in prosocial behavior

One of the questions that we addressed in this research was the role of perspective-taking skills as a possible mediator of age related differences in prosocial behavior. Indeed, we found that the age related increases in prosocial behavior toward friends was mediated by self-reported perspective taking in the *Non-costly prosocial* game. It has been shown that perspective-taking has a protracted developmental trajectory into late adolescence (Dumontheil et al., 2009). We provide further support for this developmental trajectory based on self-reported perspective-taking, and this pattern is linked to differences in prosocial behavior.

Considering that we found support for the mediating role of perspective-taking in age related increase in prosocial behavior only in one of the games examined here, caution must be taken in interpreting these results and their implications for generalization. Interestingly, only non-costly prosocial behavior was mediated by perspective-taking. Possibly, in the *Costly prosocial* game where prosocial behavior is costly, changes in other aspects of cognitive development, such as executive functioning and cognitive control, are more strongly related to costly prosocial behavior, where control of self-maximizing impulses play a role (Steinbeis et al., 2012; Luengo Kanacri et al., 2013). Although prosocial behavior in the *Disadvantageous prosocial* game was not costly, it can be considered as costly in terms of comparative interpersonal costs because it leads to a disadvantagous distribution of coins for the participant. In this sense, it could be that control of impulses also plays a relatively more important role than perspective-taking skills in this form of prosocial behavior. For future research it will be interesting to examine other interaction partners, such as parents, to better understand the aspects of social context that triggers perspective-taking and prosocial behavior. Previous studies also point out that perspective-taking skills play a significant role in both trust and reciprocity decision (Malhotra, 2004; van den Bos et al., 2010, 2011a,b). In the current study, due to practical considerations we could not employ similar study designs that would allow us to examine the role of perspective taking in trust and reciprocity. Future studies should aim to employ task manipulations that specifically address the role of perspective-taking in trust and reciprocity decision with different interaction partners.

## Concluding remarks

In the current study, we did not examine the role of gender in prosocial behavior in interactions with peers due to too small sample sizes per gender and age group. There is ample evidence on gender differences in both peer relationships and displays of prosocial behavior (Maccoby, 1986; Eisenberg et al., 1996). Across middle childhood and adolescence friendships are typically same-sex dyads, and friendships of girls are more often characterized by prosocial behavior, whereas friendships of boys more often involve displays of antisocial behavior (Güroğlu et al., 2007). Considering the relatively low prevalence of same-sex antipathy relationships (Güroğlu et al., 2009a,b), peer nominations were not restricted to same-sex nominations in the current study. Also, the small sample size within each age group did not allow us to examine gender effects. Future research should further examine the role of gender and gender combinations in peer interactions.

The experimental design of the allocation games in the current study ensured anonymity of all choices. This was done to restrict the possible role of social desirability in displays of prosocial behavior. A previous study examining sharing between friends and non-friends has shown that secret vs. public acts of sharing might differ (Buhrmester et al., 1992). Similarly, Leimgruber et al. (2012) provide evidence for strategic prosociality in 5-year-olds, where children behave more generously when the recipient is aware of the details of their actions. Considering that real-life social behavior usually takes place in the presence of others (peers, as well as parents and teachers), future studies should investigate how this aspect of context influences social decision-making across adolescence.

It is also important to note that 9-year-olds in our study do not differ in their frequency of prosocial behavior depending on the alternatives in each game. In other words, they do not differentiate between costly and non-costly prosocial behavior (see Supplementary Material). This is in contradiction with prior findings from similar and even younger age groups (Fehr et al., 2008; Blake and McAuliffe, 2011; Shaw and Olson, 2012), where decisions are shown to be affected by payoffs. Although the tasks were explained in detail to participants in small groups and all participants were given the chance to ask questions to ensure that everyone understood the task, it is possible that the youngest participants had trouble understanding the games. Future studies should include a comprehension check to assess whether children understand the payoff structure.

Here we employed a cross-sectional design to examine age differences in prosocial behavior. The current findings are highly informative for understanding developmental trajectories in prosocial behavior. Studies employing longitudinal designs are needed to reach conclusions regarding these developmental trajectories. Such longitudinal examinations will enable researchers to examine individual differences in peer relationship history (e.g., chronic rejection by peers or consistent popularity) and link these to cognitive changes (such as perspective-taking) and social behavior. However, longitudinal assessments of sociometric measures where complete school classes are tested using experimental designs as that employed here are challenging in terms of practical considerations. Future studies should focus on alternative ways of assessing prosocial behavior with real-life interaction partners that are feasible within longitudinal designs.

This study merges two important aspects of development: social decision-making and peer relationships. Our design is unique in the way it employs sociometric measures, a core method to assess peer relationships, and combines this with an experimental design using economic exchange games, which are highly efficient in examining social decision-making processes. The use of this experimental design employing allocation games tapping at different aspects of social decision-making further enabled us to examine prosocial behavior from different aspects, i.e., in terms of fairness, trust, and reciprocity considerations. The added value of this approach lies in its feasibility to examine social behavior toward different types of peers, which is not easily assessed using other methods such as questionnaire or observations of behavior. This approach is promising in understanding social exclusion in the peer context and the role of peer relationships in the treatment of bullies as well as victims (Güroğlu et al., 2013).

The differential patterns of behavior for interaction partners support the special role of friendships as forming the most significant developmental contexts across adolescence (Hartup, 1996), especially for prosocial behavior (Carlo et al., 1999). Converging evidence from all forms of behavior examined in this study is that adolescents treat friends differently than all other types of peers, and this special treatment is shaped throughout adolescence. In recent years, neuroscientific research has further highlighted the special and rewarding role of social interactions with friends (see e.g., Güroğlu et al., 2008; Braams et al., 2013). Future research needs to further pay attention to this context specificity of social behavior, and examine its links with the developing social brain.

### Conflict of interest statement

The authors declare that the research was conducted in the absence of any commercial or financial relationships that could be construed as a potential conflict of interest.

## Footnotes

^1^See Supplementary material for analyses comparing behavior across the three games per age group.
