# Supplementary material for: Sharing and giving across adolescence: an experimental study examining the development of prosocial behavior
Source: Front Psychol. 2014 Apr 11;5:291. doi: 10.3389/fpsyg.2014.00291 (PMC3990099; doi:10.3389/fpsyg.2014.00291)
Supplement: Supplementary file 1 [file Presentation1.PDF]

Supplementary material

In order to examine behavior across games, a 3 (Game) x 4 (Relationship type) repeated measures analysis of variance was conducted per age group with the frequency of prosocial offers as dependent variable. Below we report these results focusing on the role of Game; effects of Relationship type are reported in detail in the main manuscript. All analyses where the Mauchly's test indicated a violation of the assumption of sphericity, the Huyn-Feldt correction is reported.

For 9-year-olds, there were no significant effects: main effect of Game ($F(1.26,36.55) = 2.68$, $p = .11$, $\eta_p^2 = .09$), main effect of Relationship ($F(3,87) = .69$, $p = .56$, $\eta_p^2 = .02$), Game x Relationship interaction ($F(6,174) = .65$, $p = .69$, $\eta_p^2 = .02$).

For 12-year-olds, there was a main effect of Game ($F(1.43,37.16) = 8.71$, $p < .01$, $\eta_p^2 = .25$) and a main effect of Relationship ($F(2.42,62.82) = 6.16$, $p < .05$, $\eta_p^2 = .19$); the Game x Relationship interaction was not significant ($F(4.45,115.56) = .80$, $p = .38$, $\eta_p^2 = .03$). Posthoc analysis examining the Game main effect yielded that the total level of prosocial choices was significantly higher in the *Costly prosocial* game ($M = .51$, SD = .27) than in the *Non-costly prosocial* game ($M = .45$, SD = .28) and *Disadvantageous prosocial* game ($M = .28$, SD = .23), which also differed significantly from one another.

For 15-year-olds, there was a main effect of Relationship ($F(2.05,41.04) = 14.23$, $p < .001$, $\eta_p^2 = .42$); the main effect of Game ($F(1.2424.81) = 2.42$, $p = .13$, $\eta_p^2 = .11$) and the Game x Relationship interaction ($F(5.15,102.97) = .51$, $p = .78$, $\eta_p^2 = .03$) were not significant.

For 18-year-olds, there was a main effect of Game ($F(1.49,35.70) = 18.05$, $p < .001$, $\eta_p^2 = .43$) and Relationship ($F(3,72) = 26.02$, $p < .001$, $\eta_p^2 = .52$); the Game x Relationship interaction was significant at trend level ($F(6,144) = 2.10$, $p = .06$, $\eta_p^2 = .08$). The total level of prosocial behavior across the four relationship types was the highest in the *Non-costly prosocial* game ($M = .61$, $SD = .28$); prosocial behavior in the *Costly prosocial* game ($M = .50$, $SD = .29$) was also higher than in the *Disadvantageous prosocial* game ($M = .24$, $SD = .24$).
